# (*E*)-3-[5-(Di­phenyl­amino)­thio­phen-2-yl]-1-(pyridin-3-yl)prop-2-en-1-one

**DOI:** 10.1107/S1600536813021946

**Published:** 2013-08-10

**Authors:** Rui Li, Dan-Dan Li, Jie-Ying Wu

**Affiliations:** a Department of Chemistry, Anhui University, Hefei 230039, People’s Republic of China

## Abstract

In the title compound, C_24_H_18_N_2_OS, the pyridine and the two phenyl rings are oriented at dihedral angles of 10.1 (5), 71.7 (6) and 68.7 (5)°, respectively, to the central thio­phene ring. In the crystal, pairs of weak C—H⋯O hydrogen bonds link inversion-related mol­ecules, forming dimers. The dimers are linked by further weak C—H⋯O hydrogen bonds, forming chains running along the *a-*axis direction.

## Related literature

For background to the title compound, see: Wan & Mak (2011 ▶). For related compounds, see: Encinas (2002 ▶); Feng *et al.* (2012 ▶).
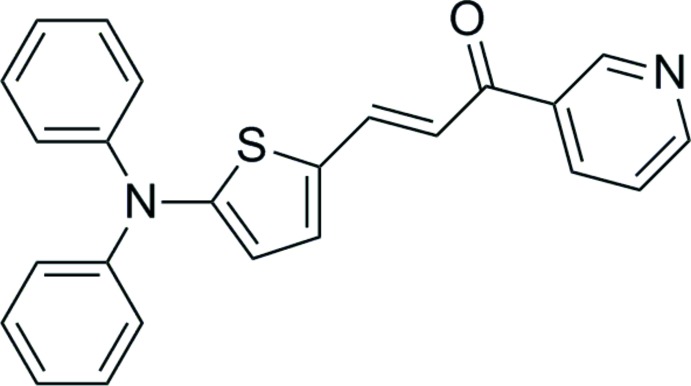


## Experimental

### 

#### Crystal data

C_24_H_18_N_2_OS
*M* *_r_* = 382.46
Monoclinic, 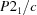

*a* = 10.976 (5) Å
*b* = 18.029 (5) Å
*c* = 9.697 (5) Å
β = 90.728 (5)°
*V* = 1918.7 (14) Å^3^
*Z* = 4
Mo *K*α radiation
μ = 0.19 mm^−1^
*T* = 293 K
0.30 × 0.20 × 0.20 mm

#### Data collection

Bruker SMART 1000 CCD area-detector diffractometer
13536 measured reflections
3388 independent reflections
2517 reflections with *I* > 2σ(*I*)
*R*_int_ = 0.031

#### Refinement

*R*[*F* ^2^ > 2σ(*F* ^2^)] = 0.039
*wR*(*F* ^2^) = 0.138
*S* = 0.95
3388 reflections
253 parameters
H-atom parameters constrained
Δρ_max_ = 0.14 e Å^−3^
Δρ_min_ = −0.29 e Å^−3^

### 

Data collection: *SMART* (Bruker, 2007 ▶); cell refinement: *SAINT* (Bruker, 2007 ▶); data reduction: *SAINT*; program(s) used to solve structure: *SHELXTL* (Sheldrick, 2008 ▶); program(s) used to refine structure: *SHELXTL*; molecular graphics: *SHELXTL*; software used to prepare material for publication: *SHELXTL*.

## Supplementary Material

Crystal structure: contains datablock(s) I, Global. DOI: 10.1107/S1600536813021946/xu5725sup1.cif

Structure factors: contains datablock(s) I. DOI: 10.1107/S1600536813021946/xu5725Isup2.hkl

Supplementary material file. DOI: 10.1107/S1600536813021946/xu5725Isup3.cml

Additional supplementary materials:  crystallographic information; 3D view; checkCIF report

## Figures and Tables

**Table 1:** Hydrogen-bond geometry (Å, °)

*D*—H⋯*A*	*D*—H	H⋯*A*	*D*⋯*A*	*D*—H⋯*A*
C11—H11⋯O1^i^	0.93	2.54	3.410 (3)	155
C12—H12⋯O1^ii^	0.93	2.43	3.346 (3)	166

Symmetry codes: (i) 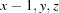; (ii) 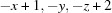.

**Fig. 1.**
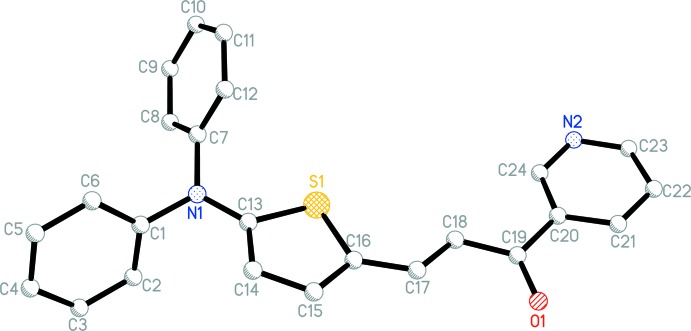
The molecular structure of the title compound (I) showing 30% probability displacement ellipsoids.

## supplementary crystallographic information

### 1. Comment

Carbonyl group widely exists in organic and biological systems and plays a crucial role in stabilizing both the extended crystal structures of small molecules and biological systems through various weak intermolecular interactions generated *via* carbonyl group (Wan & Mak, 2011). Besides, the introduction about the highpolarizability of sulfur atoms in thiophene rings leads to a stabilization of the conjugated chain and to excellent charge transport properties, which are one of the most crucial assets for applications in organic and molecular electronics (Encinas, 2002; Feng *et al.*, 2012).

The crystal structure of the title compound, exists in an E configuration with respect to the C17=C18 ethenyl bond (1.332 (3) Å), as indicated by the torsion angle C16—C17—C18—C19 = 177.90 (1) °. The prop-2-en-1-one unit (C17—C19/O1) is nearly planar and the torsion angle O1—C17—C18—C19 is 8.2 (3) °. The Carbonyl bridge is nearly planar to the pyridyl ring and the thiophene ring make the dihedral angles of 7.22 (7)° and 7.07 (8)°, respectively (Fig.1). In the terminal phenyl rings region of the molecule, each phenyl group makes dihedral angles of 71.7 (6)° and 68.7 (5)° with the thiophene ring.

### 2. Experimental

The title compound was synthesized by mixing 3-acetylpyridine (1.21 g, 10 mmol) with 5-(diphenylamino)thiophene-2-carbaldehyde (2.79 g, 10 mmol) in methanol (25 ml) in the presence of 20% NaOH (aq) (5 ml). The mixture was stirred at room temperature for 12 h. The red solid formed was filtered and washed with distilled water, dried over vacuum. 1H NMR: (400 Hz, DMSO-d^6^), d(p.p.m.): 9.05 (d, 1H), 8.48 (d, 1H), 8.25 (d, 1H), 8.12 (d, 1H), 7.86 (d, 1*H*), 7.64 (m, 1H), 7.55 (t, 4H), 7.40 (d, 1H), 7.32 (d, 4H),7.29 (t, 2H), 6.65 (d, 1H)

### 3. Refinement

All hydrogen atoms were placed in geometrically idealized positions and constrained to ride on their parent atoms, with C—H = 0.93 Å, *U*_iso_(H) = 1.2*U*_eq_(C).

### Crystal data

**Table:**

C_24_H_18_N_2_OS	*F*(000) = 800
*M**_r_* = 382.46	*D*_x_ = 1.324 Mg m^−^^3^
Monoclinic, *P*2_1_/*c*	Mo *K*α radiation, λ = 0.71069 Å
Hall symbol: -P 2ybc	Cell parameters from 3272 reflections
*a* = 10.976 (5) Å	θ = 2.2–22.8°
*b* = 18.029 (5) Å	µ = 0.19 mm^−^^1^
*c* = 9.697 (5) Å	*T* = 293 K
β = 90.728 (5)°	Block, red
*V* = 1918.7 (14) Å^3^	0.30 × 0.20 × 0.20 mm
*Z* = 4	

### Data collection

**Table:**

Bruker SMART 1000 CCD area-detector diffractometer	2517 reflections with *I* > 2σ(*I*)
Radiation source: fine-focus sealed tube	*R*_int_ = 0.031
Graphite monochromator	θ_max_ = 25.0°, θ_min_ = 1.9°
phi and ω scans	*h* = −13→13
13536 measured reflections	*k* = −21→21
3388 independent reflections	*l* = −11→11

### Refinement

**Table:**

Refinement on *F*^2^	Primary atom site location: structure-invariant direct methods
Least-squares matrix: full	Secondary atom site location: difference Fourier map
*R*[*F*^2^ > 2σ(*F*^2^)] = 0.039	Hydrogen site location: inferred from neighbouring sites
*wR*(*F*^2^) = 0.138	H-atom parameters constrained
*S* = 0.95	*w* = 1/[σ^2^(*F*_o_^2^) + (0.1*P*)^2^] where *P* = (*F*_o_^2^ + 2*F*_c_^2^)/3
3388 reflections	(Δ/σ)_max_ < 0.001
253 parameters	Δρ_max_ = 0.14 e Å^−^^3^
0 restraints	Δρ_min_ = −0.29 e Å^−^^3^

### Special details

**Table:**

Geometry. All e.s.d.'s (except the e.s.d. in the dihedral angle between two l.s. planes) are estimated using the full covariance matrix. The cell e.s.d.'s are taken into account individually in the estimation of e.s.d.'s in distances, angles and torsion angles; correlations between e.s.d.'s in cell parameters are only used when they are defined by crystal symmetry. An approximate (isotropic) treatment of cell e.s.d.'s is used for estimating e.s.d.'s involving l.s. planes.
Refinement. Refinement of *F*^2^ against ALL reflections. The weighted *R*-factor *wR* and goodness of fit *S* are based on *F*^2^, conventional *R*-factors *R* are based on *F*, with *F* set to zero for negative *F*^2^. The threshold expression of *F*^2^ > σ(*F*^2^) is used only for calculating *R*-factors(gt) *etc*. and is not relevant to the choice of reflections for refinement. *R*-factors based on *F*^2^ are statistically about twice as large as those based on *F*, and *R*- factors based on ALL data will be even larger.

### Fractional atomic coordinates and isotropic or equivalent isotropic displacement parameters (Å^2^)

**Table:**

	*x*	*y*	*z*	*U*_iso_*/*U*_eq_	
S1	0.39364 (5)	0.13546 (3)	0.84763 (5)	0.0520 (2)	
O1	0.80534 (14)	0.06367 (10)	1.13966 (15)	0.0628 (4)	
N1	0.23384 (15)	0.12744 (10)	0.63004 (17)	0.0518 (5)	
C18	0.61524 (18)	0.11137 (11)	1.0700 (2)	0.0471 (5)	
H18	0.5526	0.1439	1.0917	0.057*	
C14	0.41913 (19)	0.04995 (12)	0.6381 (2)	0.0483 (5)	
H14	0.4065	0.0256	0.5546	0.058*	
C7	0.13189 (17)	0.15100 (11)	0.70872 (19)	0.0438 (5)	
C20	0.70392 (18)	0.12875 (11)	1.3133 (2)	0.0431 (5)	
C13	0.33940 (17)	0.10074 (11)	0.69095 (19)	0.0428 (5)	
C15	0.52143 (18)	0.03859 (11)	0.7228 (2)	0.0480 (5)	
H15	0.5835	0.0058	0.7003	0.058*	
C19	0.71424 (18)	0.09932 (11)	1.1691 (2)	0.0448 (5)	
C16	0.52275 (17)	0.07951 (11)	0.8412 (2)	0.0428 (5)	
C1	0.22037 (18)	0.12040 (11)	0.4829 (2)	0.0444 (5)	
C2	0.3006 (2)	0.15559 (14)	0.3977 (2)	0.0576 (6)	
H2	0.3623	0.1851	0.4348	0.069*	
C12	0.08974 (19)	0.10913 (13)	0.8175 (2)	0.0511 (5)	
H12	0.1278	0.0647	0.8410	0.061*	
C17	0.61310 (18)	0.07655 (11)	0.9487 (2)	0.0445 (5)	
H17	0.6796	0.0461	0.9316	0.053*	
N2	0.60796 (19)	0.20514 (11)	1.4845 (2)	0.0666 (6)	
C8	0.0729 (2)	0.21623 (12)	0.6732 (2)	0.0561 (6)	
H8	0.1001	0.2440	0.5988	0.067*	
C11	−0.0099 (2)	0.13381 (15)	0.8915 (2)	0.0644 (7)	
H11	−0.0384	0.1059	0.9650	0.077*	
C21	0.7850 (2)	0.10299 (13)	1.4133 (2)	0.0559 (6)	
H21	0.8449	0.0689	1.3901	0.067*	
C6	0.1268 (2)	0.07803 (13)	0.4276 (3)	0.0601 (6)	
H6	0.0709	0.0552	0.4850	0.072*	
C24	0.61859 (19)	0.17992 (12)	1.3549 (2)	0.0541 (5)	
H24	0.5646	0.1983	1.2885	0.065*	
C9	−0.0253 (2)	0.24017 (14)	0.7469 (3)	0.0668 (7)	
H9	−0.0641	0.2842	0.7225	0.080*	
C22	0.7765 (3)	0.12783 (14)	1.5463 (3)	0.0663 (7)	
H22	0.8298	0.1110	1.6147	0.080*	
C10	−0.0670 (2)	0.19950 (16)	0.8567 (3)	0.0686 (7)	
H10	−0.1331	0.2162	0.9071	0.082*	
C5	0.1175 (3)	0.07000 (15)	0.2857 (3)	0.0767 (8)	
H5	0.0553	0.0412	0.2476	0.092*	
C4	0.1995 (3)	0.10438 (18)	0.2009 (3)	0.0751 (8)	
H4	0.1934	0.0985	0.1058	0.090*	
C3	0.2895 (2)	0.14712 (17)	0.2569 (2)	0.0729 (8)	
H3	0.3443	0.1709	0.1993	0.087*	
C23	0.6874 (2)	0.17810 (14)	1.5755 (3)	0.0675 (7)	
H23	0.6821	0.1946	1.6660	0.081*	

### Atomic displacement parameters (Å^2^)

**Table:**

	*U*^11^	*U*^22^	*U*^33^	*U*^12^	*U*^13^	*U*^23^
S1	0.0452 (4)	0.0707 (4)	0.0401 (3)	0.0160 (3)	−0.0087 (2)	−0.0122 (2)
O1	0.0445 (9)	0.0911 (12)	0.0526 (10)	0.0187 (8)	−0.0041 (7)	−0.0018 (8)
N1	0.0342 (9)	0.0881 (13)	0.0330 (9)	0.0109 (9)	−0.0021 (7)	−0.0039 (8)
C18	0.0383 (12)	0.0567 (12)	0.0462 (13)	0.0059 (9)	−0.0039 (9)	0.0020 (9)
C14	0.0432 (12)	0.0627 (13)	0.0389 (11)	0.0028 (9)	−0.0020 (9)	−0.0086 (9)
C7	0.0338 (11)	0.0625 (13)	0.0349 (11)	0.0022 (9)	−0.0034 (8)	−0.0073 (9)
C20	0.0379 (11)	0.0476 (11)	0.0438 (11)	−0.0084 (9)	−0.0041 (9)	0.0020 (9)
C13	0.0345 (11)	0.0594 (12)	0.0346 (11)	0.0007 (9)	−0.0002 (8)	0.0002 (9)
C15	0.0419 (12)	0.0579 (12)	0.0441 (12)	0.0104 (9)	0.0013 (9)	−0.0041 (9)
C19	0.0366 (11)	0.0519 (11)	0.0457 (12)	−0.0003 (9)	−0.0013 (9)	0.0059 (9)
C16	0.0364 (11)	0.0524 (11)	0.0396 (11)	0.0035 (8)	−0.0020 (9)	0.0011 (9)
C1	0.0374 (11)	0.0597 (12)	0.0360 (11)	0.0080 (9)	−0.0041 (9)	−0.0016 (9)
C2	0.0415 (12)	0.0846 (16)	0.0466 (13)	−0.0002 (11)	−0.0008 (10)	0.0028 (11)
C12	0.0446 (12)	0.0659 (13)	0.0426 (12)	0.0064 (10)	−0.0030 (9)	−0.0026 (10)
C17	0.0363 (11)	0.0527 (12)	0.0444 (12)	0.0044 (9)	−0.0013 (9)	0.0060 (9)
N2	0.0664 (14)	0.0705 (13)	0.0630 (14)	−0.0058 (10)	0.0013 (11)	−0.0172 (10)
C8	0.0509 (13)	0.0625 (14)	0.0547 (13)	0.0030 (11)	−0.0055 (10)	−0.0002 (10)
C11	0.0512 (15)	0.1042 (19)	0.0378 (12)	−0.0055 (13)	0.0062 (11)	−0.0059 (12)
C21	0.0513 (13)	0.0656 (14)	0.0504 (14)	0.0010 (11)	−0.0105 (10)	−0.0023 (11)
C6	0.0519 (13)	0.0729 (15)	0.0551 (14)	−0.0091 (11)	−0.0102 (11)	0.0015 (11)
C24	0.0448 (12)	0.0574 (13)	0.0600 (15)	−0.0023 (10)	−0.0046 (10)	−0.0020 (11)
C9	0.0579 (15)	0.0697 (15)	0.0725 (17)	0.0199 (12)	−0.0076 (13)	−0.0161 (13)
C22	0.0708 (17)	0.0762 (16)	0.0513 (15)	−0.0045 (13)	−0.0187 (12)	−0.0019 (12)
C10	0.0450 (13)	0.104 (2)	0.0563 (15)	0.0204 (13)	−0.0027 (11)	−0.0312 (14)
C5	0.0746 (18)	0.0893 (19)	0.0654 (17)	0.0030 (15)	−0.0307 (15)	−0.0209 (14)
C4	0.0682 (18)	0.119 (2)	0.0377 (13)	0.0341 (16)	−0.0075 (13)	−0.0104 (14)
C3	0.0517 (15)	0.123 (2)	0.0440 (14)	0.0178 (14)	0.0038 (12)	0.0117 (14)
C23	0.0771 (18)	0.0750 (16)	0.0503 (14)	−0.0212 (14)	0.0000 (13)	−0.0149 (12)

### Geometric parameters (Å, º)

**Table:**

S1—C13	1.741 (2)	C12—H12	0.9300
S1—C16	1.741 (2)	C17—H17	0.9300
O1—C19	1.226 (2)	N2—C23	1.325 (3)
N1—C13	1.380 (3)	N2—C24	1.343 (3)
N1—C7	1.427 (2)	C8—C9	1.371 (3)
N1—C1	1.439 (3)	C8—H8	0.9300
C18—C17	1.333 (3)	C11—C10	1.380 (4)
C18—C19	1.458 (3)	C11—H11	0.9300
C18—H18	0.9300	C21—C22	1.369 (3)
C14—C13	1.371 (3)	C21—H21	0.9300
C14—C15	1.398 (3)	C6—C5	1.387 (4)
C14—H14	0.9300	C6—H6	0.9300
C7—C12	1.382 (3)	C24—H24	0.9300
C7—C8	1.384 (3)	C9—C10	1.376 (4)
C20—C24	1.378 (3)	C9—H9	0.9300
C20—C21	1.387 (3)	C22—C23	1.365 (4)
C20—C19	1.501 (3)	C22—H22	0.9300
C15—C16	1.365 (3)	C10—H10	0.9300
C15—H15	0.9300	C5—C4	1.374 (4)
C16—C17	1.431 (3)	C5—H5	0.9300
C1—C2	1.371 (3)	C4—C3	1.361 (4)
C1—C6	1.382 (3)	C4—H4	0.9300
C2—C3	1.378 (3)	C3—H3	0.9300
C2—H2	0.9300	C23—H23	0.9300
C12—C11	1.389 (3)		
			
C13—S1—C16	91.72 (9)	C16—C17—H17	115.5
C13—N1—C7	122.32 (17)	C23—N2—C24	115.7 (2)
C13—N1—C1	117.96 (16)	C9—C8—C7	120.4 (2)
C7—N1—C1	119.03 (16)	C9—C8—H8	119.8
C17—C18—C19	121.08 (19)	C7—C8—H8	119.8
C17—C18—H18	119.5	C10—C11—C12	120.4 (2)
C19—C18—H18	119.5	C10—C11—H11	119.8
C13—C14—C15	112.96 (18)	C12—C11—H11	119.8
C13—C14—H14	123.5	C22—C21—C20	119.9 (2)
C15—C14—H14	123.5	C22—C21—H21	120.1
C12—C7—C8	119.63 (19)	C20—C21—H21	120.1
C12—C7—N1	121.20 (19)	C1—C6—C5	119.2 (2)
C8—C7—N1	119.16 (18)	C1—C6—H6	120.4
C24—C20—C21	116.9 (2)	C5—C6—H6	120.4
C24—C20—C19	124.62 (19)	N2—C24—C20	124.6 (2)
C21—C20—C19	118.52 (19)	N2—C24—H24	117.7
C14—C13—N1	127.55 (19)	C20—C24—H24	117.7
C14—C13—S1	110.71 (15)	C8—C9—C10	120.5 (2)
N1—C13—S1	121.59 (15)	C8—C9—H9	119.8
C16—C15—C14	114.53 (18)	C10—C9—H9	119.8
C16—C15—H15	122.7	C23—C22—C21	118.0 (2)
C14—C15—H15	122.7	C23—C22—H22	121.0
O1—C19—C18	121.86 (19)	C21—C22—H22	121.0
O1—C19—C20	118.29 (18)	C9—C10—C11	119.5 (2)
C18—C19—C20	119.82 (18)	C9—C10—H10	120.2
C15—C16—C17	126.36 (19)	C11—C10—H10	120.2
C15—C16—S1	110.08 (15)	C4—C5—C6	120.4 (2)
C17—C16—S1	123.53 (15)	C4—C5—H5	119.8
C2—C1—C6	120.1 (2)	C6—C5—H5	119.8
C2—C1—N1	119.88 (19)	C3—C4—C5	119.7 (2)
C6—C1—N1	119.98 (19)	C3—C4—H4	120.2
C1—C2—C3	119.8 (2)	C5—C4—H4	120.2
C1—C2—H2	120.1	C4—C3—C2	120.8 (2)
C3—C2—H2	120.1	C4—C3—H3	119.6
C7—C12—C11	119.5 (2)	C2—C3—H3	119.6
C7—C12—H12	120.2	N2—C23—C22	125.0 (2)
C11—C12—H12	120.2	N2—C23—H23	117.5
C18—C17—C16	129.0 (2)	C22—C23—H23	117.5
C18—C17—H17	115.5		
			
C13—N1—C7—C12	−45.5 (3)	C6—C1—C2—C3	−1.6 (3)
C1—N1—C7—C12	124.8 (2)	N1—C1—C2—C3	178.2 (2)
C13—N1—C7—C8	135.9 (2)	C8—C7—C12—C11	−1.2 (3)
C1—N1—C7—C8	−53.9 (3)	N1—C7—C12—C11	−179.87 (19)
C15—C14—C13—N1	176.09 (19)	C19—C18—C17—C16	177.90 (19)
C15—C14—C13—S1	0.6 (2)	C15—C16—C17—C18	−175.2 (2)
C7—N1—C13—C14	150.3 (2)	S1—C16—C17—C18	2.6 (3)
C1—N1—C13—C14	−20.1 (3)	C12—C7—C8—C9	1.3 (3)
C7—N1—C13—S1	−34.7 (3)	N1—C7—C8—C9	179.96 (19)
C1—N1—C13—S1	154.93 (15)	C7—C12—C11—C10	0.2 (3)
C16—S1—C13—C14	−0.95 (16)	C24—C20—C21—C22	0.9 (3)
C16—S1—C13—N1	−176.74 (17)	C19—C20—C21—C22	−178.9 (2)
C13—C14—C15—C16	0.2 (3)	C2—C1—C6—C5	1.8 (3)
C17—C18—C19—O1	8.2 (3)	N1—C1—C6—C5	−178.0 (2)
C17—C18—C19—C20	−169.57 (19)	C23—N2—C24—C20	0.6 (3)
C24—C20—C19—O1	168.2 (2)	C21—C20—C24—N2	−1.1 (3)
C21—C20—C19—O1	−12.1 (3)	C19—C20—C24—N2	178.65 (19)
C24—C20—C19—C18	−13.9 (3)	C7—C8—C9—C10	−0.3 (4)
C21—C20—C19—C18	165.80 (19)	C20—C21—C22—C23	−0.3 (4)
C14—C15—C16—C17	177.10 (19)	C8—C9—C10—C11	−0.7 (4)
C14—C15—C16—S1	−0.9 (2)	C12—C11—C10—C9	0.8 (4)
C13—S1—C16—C15	1.05 (16)	C1—C6—C5—C4	−0.7 (4)
C13—S1—C16—C17	−177.03 (18)	C6—C5—C4—C3	−0.7 (4)
C13—N1—C1—C2	−62.3 (3)	C5—C4—C3—C2	0.9 (4)
C7—N1—C1—C2	127.0 (2)	C1—C2—C3—C4	0.2 (4)
C13—N1—C1—C6	117.6 (2)	C24—N2—C23—C22	0.0 (4)
C7—N1—C1—C6	−53.1 (3)	C21—C22—C23—N2	−0.2 (4)

### Hydrogen-bond geometry (Å, º)

**Table:**

*D*—H···*A*	*D*—H	H···*A*	*D*···*A*	*D*—H···*A*
C11—H11···O1^i^	0.93	2.54	3.410 (3)	155
C12—H12···O1^ii^	0.93	2.43	3.346 (3)	166

Symmetry codes: (i) *x*−1, *y*, *z*; (ii) −*x*+1, −*y*, −*z*+2.
